# Adaptation of Conductometric Monoenzyme Biosensor for Rapid Quantitative Analysis of L-arginine in Dietary Supplements

**DOI:** 10.3390/s24144672

**Published:** 2024-07-18

**Authors:** Olga Y. Saiapina, Kseniia Berketa, Andrii S. Sverstiuk, Lyubov Fayura, Andriy A. Sibirny, Sergei Dzyadevych, Oleksandr O. Soldatkin

**Affiliations:** 1 Institute of Molecular Biology and Genetics, National Academy of Sciences of Ukraine, 150 Zabolotnyi Str., 03680 Kyiv, Ukraine; osayapina4@gmail.com (O.Y.S.); s.v.dzyadevych@imbg.org.ua (S.D.); o.o.soldatkin@imbg.org.ua (O.O.S.); 2 Institute of Biology and Medicine, Taras Shevchenko National University of Kyiv, Volodymyrska Street 64, 01003 Kyiv, Ukraine; 3 Department of Medical Informatics, I. Horbachevsky Ternopil National Medical University, Maidan Voli Str., 1, 46002 Ternopil, Ukraine; 4 Department of Computer Sciences, Ternopil National Ivan Puluj Technical University, Rus’ka Str., 56, 46001 Ternopil, Ukraine; 5 Institute of Cell Biology, National Academy of Science of Ukraine, 14/16 Drahomanov Str., 79005 Lviv, Ukraine; fayura@cellbiol.lviv.ua (L.F.); sibirny@cellbiol.lviv.ua (A.A.S.); 6 Department of Biotechnology and Microbiology, Rzeszow University, Zelwerowicza 4, 35-601 Rzeszow, Poland; 7 Igor Sikorsky Kyiv Polytechnic Institute, Beresteyskyi ave. 37, 03056 Kyiv, Ukraine

**Keywords:** arginine, monoenzyme biosensor, arginine deiminase, conductometric transducer, real sample analysis

## Abstract

The present study reports on the development, adaptation, and optimization of a novel monoenzyme conductometric biosensor based on a recombinant arginine deiminase (ADI) for the determination of arginine in dietary supplements with a high accuracy of results. Aiming for the highly sensitive determination of arginine in real samples, we studied the effect of parameters of the working buffer solution (its pH, buffer capacity, ionic strength, temperature, and protein concentration) on the sensitivity of the biosensor to arginine. Thus, it was determined that the optimal buffer is a 5 mM phosphate buffer solution with pH 6.2, and the optimal temperature is 39.5 °C. The linear functioning range is 2.5–750 µM of L-arginine with a minimal limit of detection of 2 µM. The concentration of arginine in food additive samples was determined using the developed ADI-based biosensor. Based on the obtained results, the most effective method of biosensor analysis using the method of standard additions was chosen. It was also checked how the reproducibility of the biosensor changes during the analysis of pharmaceutical samples. The results of the determination of arginine in real samples using a conductometric biosensor based on ADI clearly correlated with the data obtained using the method of ion-exchange chromatography and enzymatic spectrophotometric analysis. We concluded that the developed biosensor would be effective for the accurate and selective determination of arginine in dietary supplements intended for the prevention and/or elimination of arginine deficiency.

## 1. Introduction

Today, the treatment and prevention of the deficiency of key nutrients and biologically active substances in the human body is important for the restoration of the basic functions of the body and therefore should be increasingly used for therapeutic and rehabilitation purposes in medicine. One of the compounds that plays a special role in a number of synthetic processes in the body is the conditionally indispensable amino acid L-arginine. It is known that in the body of a healthy person, arginine can be synthesized from L-glutamine, while in the body of premature children, the elderly, and in some conditions and diseases, it is not produced or is produced in insufficient quantities [1,2].

In the human body, arginine is a component of peptides and proteins, acts as a precursor of urea, proline, glutamate, creatine, agmatine, L-ornithine, L-citrulline, and γ-aminobutyric acid, and is the only precursor of nitric oxide NO. It is important to note that arginine deficiency is a key stage in the series of mechanisms that lead to the development of endothelial dysfunction and NO deficiency in a number of pathologies. That is why medicinal products and dietary supplements based on L-arginine are widely used today for the prevention and treatment of all manifestations of endothelial dysfunction of an atherosclerotic origin (cardiac, cerebral, peripheral), hypertension (arterial, pulmonary, renal), liver and skin diseases, immunodeficiency states, osteoarthritis [3,4,5,6,7,8], and in post-traumatic and postoperative periods for the rapid healing of wounds, burns, and injuries. Thus, the possibility of carrying out the accurate, selective, and at the same time rapid determination of the concentration of arginine in the amino acid profile of the product is relevant to ensure the proper control of food and medicine quality. In turn, this can become the basis for the implementation of a simplified procedure for the quality control of products designed to support health and/or satisfy the body’s acute need for arginine.

The content of L-arginine varies from product to product. The most arginine-rich products are soy beans or flowers (2.4–2.9 g of L-arginine per 100 g of product), sunflower seeds (2.0 g/100 g), sesame seeds (2.2 g/100 g), different nuts, meat (1.4–1.8 g/100 g, depending on the kind of meat), and others [9]. The usual content of L-arginine in supplements is between 250 to 1000 mg per tablet or capsule, depending on the manufacturer and the purpose of the medicine [10,11].

Today, the most common methods for determining arginine in laboratory practice are instrumental methods, such as ion-exchange and high-performance liquid chromatography [12,13], fluorimetric [14], chemiluminescence [15], and spectrophotometric analysis [16,17], capillary electrophoresis [18], and mass spectrometry [19]. Enzymatic methods are a separate group of methods that are also widely used [20,21,22,23]. The enzymatic methods of analysis described today are most often based on one or more reactions of arginine conversion with the participation of arginase, urease, and glutamate dehydrogenase [20], arginine deiminase, argininosuccinate synthase, pyruvate phosphate dikinase, pyruvate oxidase, and horseradish peroxidase [22], arginase and urease [23], or arginine deiminases [21].

Despite good analytical characteristics, the use of instrumental methods in the analysis of multicomponent samples, such as food products and dietary supplements, often requires lengthy sample preparation, the presence of highly qualified personnel, the use of expensive reagents, and expensive and bulky equipment. Enzymatic methods, despite their relative simplicity, are performed in offline mode using photosensitive or unstable chromogenic reagents and fluorophores, which complicates their widespread implementation. Considering the above, the transition to fast, small-sized, and at the same time reliable and relatively cheap methods and analytical devices for the determination of arginine is a rather relevant direction in modern laboratory practice.

As previously shown, methods of analysis that use the properties of biomaterials in combination with electrochemical, optical, or other signal measurement systems have great prospects for overcoming the challenges that modern science must answer [24]. Such methods include electrochemical biosensors, which, compared to other types of sensor systems, are characterized by a relatively simple manufacturing and use technology, speed of analysis and processing of results, and a wide range of substances that can be determined.

Biosensors for determining the function of arginine are known today based on one or more arginine-decomposing enzymes (arginine deaminases, arginases, ureases, L-arginine decarboxylases), ammonium-sensitive nanoparticles, and various types of physical transducers (amperometric or conductometric transducers, pH-sensitive field-effect transistors and ion-selective electrodes) [25,26,27].

Conductometric biosensors, compared to other types (amperometric or potentiometric), are easier to use, because, unlike others, they do not require the use of reference or auxiliary electrodes which makes the measurement process simpler, more cost-efficient, portable, and easy-to-use. Also, the usage of differential measurement modes in our case helps to reduce the effect of non-specific reactions of the biosensor, which can occur through some uninformative changes in the environment or due to the presence of additional substances in real samples that are not the target analyte [28].

Most of the biosensors for arginine described in the literature show a limited potential for the real analysis of samples and, in addition, are based on the use of a bienzymatic selective element [29,30,31,32,33,34]. Considering the advantages of single-enzyme biosensors compared to multi-enzyme ones (much higher or absolute selectivity to the target analyte, lower cost, and simpler manufacturing procedure of bioselective elements), it is clear that the use of single-enzyme biosensors for the analysis of real samples is more appropriate and relevant.

In this work, a new conductometric biosensor was developed for the determination of L-arginine based on recombinant arginine deiminase (ADI), which decomposes arginine according to the following reaction [35,36]:

ADI (EC 3.5.3.6) L-arginine + H_2_O + H^+^ → L-citrulline + NH_4_^+^ (1)

The main measured parameter of the biosensor is the change in the difference between the output active conductivity of two pairs of electrodes (ΔG) of the conductometric transducer due to the appearance, change in concentration, and mobility of ions in the solution during the breakdown of the arginine molecule.

The highly stable ADI enzyme from *Mycoplasma hominis* was used for the development of the biosensor, which was obtained on the basis of a modified protocol [37]. The work used an enzyme preparation, in which a recombinant E. coli strain was constructed that overexpresses the *M. hominis* gene encoding ADI. The obtained enzyme preparation was characterized by high specific activity for 5 years when stored at +4 °C.

The work aimed to carry out a series of optimizations aimed at developing a highly sensitive biosensor based on ADI, which could be used in the future for the determination of L-arginine in real samples. The procedure for optimizing the operation of the biosensor included the analysis of parameters of the optimal buffer solution for the operation of the biosensor. In the buffer solution of the optimized composition, analytical characteristics were investigated, and its selectivity, signal reproducibility, and storage stability were evaluated.

The analysis of the concentration of arginine in dietary supplements was carried out using a newly developed biosensor, taking into account the possibility of the influence of the preliminary preparation of the sample on the accuracy of the results during the measurement. During this study, spectrophotometry and ion chromatography were used as control methods (the latter method was performed by an independent laboratory).

The results obtained as part of the work were carefully studied and analyzed, as well as an assessment of the prospects of using such a biosensor to determine the concentration of arginine in pharmaceutical samples.

## 2. Materials and Methods

### 2.1. Materials

For this work, we used a preparation of recombinant arginine deiminase (EC 3.5.3.6) from Mycoplasma hominis, expressed in Escherichia coli cells, with a volume activity of 147 U/mL, bovine serum albumin (BSA), fraction V, 25% aqueous solution of glutaraldehyde (GA), L-valine, L-serine, L-glycine, L-proline, L-threonine, L-histidine, L-asparagine, L-tryptophan, and L-ascorbic acid, which were purchased from Sigma-Aldrich (St. Louis, MO, USA), and L-arginine, L-arginine monohydrochloride, and L-glutamate, from Sigma-Aldrich (Steinheim, Germany); L-glutamine and L-isoleucine were from Sigma-Aldrich (São Paulo, Brazil), L-cysteine, L-methionine, and L-phenylalanine were from Sigma-Aldrich (Tokyo, Japan), L-alanine and L-lysine were from Sigma-Aldrich (Shanghai, China), and citric acid was purchased from Sigma-Aldrich (Vienna, Austria). Glycerol was produced by “Macrohim” (Kyiv, Ukraine). NaCl was obtained from “Sigma-Aldrich” (USA). Different dietary supplements (“L-arginine” capsules from Solgar Inc. (Leonia, NJ, USA), “L-arginine” capsules from Now Foods Corp. (Bloomingdale, IL, USA), and “L-arginine” capsules from Elit-Pharm LLC (Dnipro, Ukraine) were purchased in the local stores.

A one-component phosphate buffer solution of the composition was used as a medium for measurements of KH_2_PO_4_. For the pH influence investigation, 2.5 mM Polymix buffer solution was used. It consists of equal concentrations of KH_2_PO_4_, tris-HCl, sodium tetraborate, and citric acid. All chemicals used in the work were of reagent grade purity. Distilled or ultrapure water was used to prepare the solutions.

### 2.2. Design of Conductometric Transducers and Measurement Equipment for Biosensor Analysis

**Figure 1:**
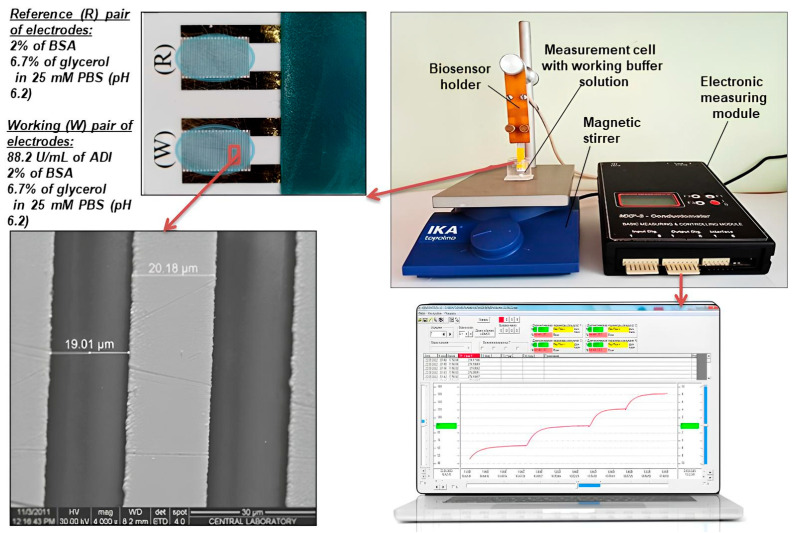
Portable conductometric analyzer used for the biosensor measurements and typical conductometric transducer.

As physical transducers, we used conductometric transducers manufactured following our recommendations at the V.E. Lashkaryov Institute of Semiconductor Physics (Kyiv, Ukraine). Each of the transducers consisted of two pairs of thin-layer planar comb electrodes made via the vacuum sputtering of gold on a non-conductive substrate made of sital (geometric dimensions of the substrate, 5 × 30 mm). To improve the adhesion of the gold layer to the sital, an auxiliary layer of chromium with a thickness of 50 nm was applied. The transducer electrodes had a counter-pin geometry with a gold layer thickness of about 150 nm. The area of the sensitive surface of a pair of electrodes was about 3 mm^2^, and the distance between the fingers of the combs and the width of the fingers themselves was about 20 μm. The transducers were used in the differential measurement mode to record changes in the conductivity of the solution layer and molecular structures located in the near-electrode region (Figure 1).

During the manufacture of the biosensor, the sensitive surface of one pair of electrodes was used to apply the working membrane based on ADI (working pair of electrodes W), and the sensitive surface of the other pair was used to apply the reference membrane based on BSA (reference pair of electrodes R).

Biosensor measurements were carried out using two types of experimental conductometric installations, namely portable and stationary installations for conductometric measurements.

The portable device for conductometric measurements consisted of a differential conductometer developed in cooperation with the Institute of Electrodynamics of the National Academy of Sciences of Ukraine (Kyiv), an electrode holder, connecting elements for connecting all system components, a computer, and software. In such a system, the conductometric transducers were connected to the compensating bridge circuit of an alternating current with a voltage of 14 mV and a frequency of 66 kHz. The block diagram of the compensation bridge circuit of the differential conductometer and a detailed description of the device are given in [38]. The appearance of the conductometric installation is presented in Figure 1.

The study of the influence of the parameters of the working solution on the functioning of the biosensor was performed on a stationary experimental setup for conductometric measurements. In earlier work by our department, this setup was widely used in the development of various enzyme biosensors [39]. An alternating voltage with an amplitude of 10 mV and a frequency of 100 kHz is applied to two pairs of intedigitated electrodes of the conductometric transducer from the G3-118 signal generator. The differential mode of measurements was used to increase the sensitivity of the sensors and reduce the noise caused by non-specific influences. During operation, the electrodes were in the measuring cell with the investigated solution. The signal received at the electrodes from the load resistors, Rn = 1 kΩ, is sent through the differential amplifier “Unipan-233-6” to the selective nanovoltmeter “Unipan-233”, from which the signal is fed directly to the recording device (recorder or personal computer). The principle of its operation was the basis for the creation of both a portable device for conductometric measurements and the differential conductometer described above. The scheme of this installation can be found in more detail in previous works [39].

### 2.3. Bioselective Membrane Preparation

The working membrane and the reference membrane of the biosensor were made using the method of cross-linking with a bifunctional agent, glutaraldehyde. To prepare the working membrane of the biosensor, a solution of arginine deiminase with a concentration of 88.2 U/mL and a solution of BSA (mass quotient in the solution, 2%) were homogenized in 25 mM phosphate buffer solution (pH 6.2) containing 6.7% glycerol. The solution for the reference membrane preparation contained the same total amount of protein as the solution for the working membrane, but the part of ADI was replaced by BSA. Each of these solutions was applied to the sensitive surface of one of the pairs of conductometric transducer electrodes and distributed on it until a homogeneous thin layer was formed. Immobilization was carried out in a desiccator in saturated vapors of glutaraldehyde. The immobilization time was 30 min. After immobilization, the biosensor was dried in air for 10 min and then washed in fresh portions of phosphate buffer solution for 10–15 min to remove unbound components of biomembranes. Between measurements, the biosensor was stored in a dry state at +4 °C.

### 2.4. Determination of Arginine in Dietary Supplements

#### 2.4.1. Procedure of the Biosensor Measurements

Measurements were performed at room temperature in an open measuring cell (2 mL) filled with a working buffer solution (5 mM phosphate buffer solution, pH 6.2) with constant stirring. The required concentration of the model solution of arginine in the cell for measurements was obtained by adding aliquots of the stock model solution of arginine to the working buffer solution. Nonspecific changes in the output signal associated with local changes in temperature, pH, and random electrical currents were eliminated by using the differential measurement mode.

To measure the concentration of L-arginine in real samples, three methods of determining unknown concentrations of L-arginine were used and compared: the classical biosensor method using a calibration curve, the method of standard additions, and the simplified method of analysis by proportion.

When using the calibration curve method, the calibration curve of the biosensor was first obtained, and its linear range was determined. Based on the concentration of arginine, which corresponded to the linear operational range of the biosensor, the dimension of sample dilution in the cell was selected. In the course of the experiment, we received feedback response on several different dilutions of solutions of real samples to establish such a dimension of the dilution at which the response of the biosensor would be in the linear section of the calibration curve. Biosensor responses were obtained for the same aliquot of the sample in several repetitions (n = 3…5), the average value was calculated, and the curve equation (y = a + b × x) corresponding to the linear operational range of the calibration curve was obtained. The concentrations of arginine in measurement cells and then (taking into account the degree of dilution of the sample) in the original solution were calculated.

When using the method of standard additions, the response of the biosensor was first obtained based on an aliquot of the sample and then on several aliquots of a model solution of L-arginine of a known concentration (three subsequent additions). In this way, four consecutive responses were obtained, from which a graph was built in such a way that the response to the studied sample was set on the Oy axis. A linear extrapolation of this graph intersected the Ox axis at a point corresponding to the concentration of L-arginine in the measurement cell. According to the degree of dilution of the sample in the measuring cell, the concentration of L-arginine in the sample was obtained.

Using a simplified ratio analysis method, we first obtained the response of the biosensor to the addition of an aliquot of the sample and then added one aliquot of the L-arginine stock solution. Next, we compared the received answers by proportions and calculated the concentration of L-arginine in the sample, taking into account its dilution. This method is the simplest of the previous ones, but its accuracy is often not sufficient.

#### 2.4.2. Reference Methods for Determining Arginine in Dietary Supplements

Two control methods were used in parallel with the biosensor analysis for the determination arginine in dietary supplements, namely spectrophotometric analysis and ion chromatography. To determine the concentration of arginine in real samples using the enzymatic spectrophotometric method, we used the L-arginine Assay Kit (Sigma Aldrich, Germany), which included a buffer for analysis, an enzyme mixture, a mixture of samples A, a mixture of samples B, a mixture for sample cleaning, and the arginine standard solution. The analysis was performed according to the protocol provided by the manufacturer.

Quantitative determination in the studied samples using the chromatography method was carried out by the independent laboratory of the “Expert Center for Diagnostics and Laboratory Support “Biolights”” LLC (Kyiv, Ukraine). The method of ion-exchange chromatography with a cation-exchange column and post-column derivatization with ninhydrin was used. The determination was made at 570 nm. The derivatization temperature was 130 °C.

Standard methods of variational statistics were used to process the experimental data obtained during the work. Studies were performed using at least 3–5 replicates. In the statistical processing of the results, the arithmetic mean and its standard deviation were determined, and the data were considered significant at *p* < 0.05. Data processing and calculations were performed using the OriginLab graphic editor (OriginPro version 8.5).

## 3. Results and Discussion

### 3.1. Effect of Changing the Parameters of the Working Buffer Solution (Analyzed Medium) on the Functioning of a Biosensor Based on Arginine Deiminase

**Figure 2:**
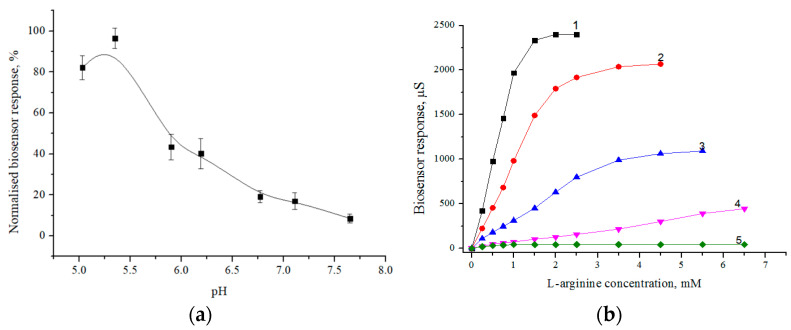
Dependence of biosensor responses on pH (**a**) and buffer capacity (**b**) of the working buffer solution. A: The concentration of L-arginine is 1.5 mM. The study of the effect of pH was carried out in 2.5 mM Polymix buffer solution. B: 1—1 mM buffer, 2—2.5 mM buffer, 3—5 mM buffer, 4—10 mM buffer, 5—20 mM buffer. The analysis of the effect of buffer capacity was carried out in phosphate buffer with pH 6.2.

It is known that the functioning of any biosensor depends not only on the characteristics of the immobilized biomaterial in the membranes of the biosensor but also on the parameters of the solution in which the measurements are performed. Of course, real samples can be characterized by different parameters of the solution. Therefore, it was necessary to conduct a study of the influence of the parameters of the buffer solution (pH, buffer capacity, and ionic strength) on the operation of the developed biosensor. Since the enzyme activity usually depends on pH, in order to more accurately determine the pH-optimum of the developed biosensor, the pH-dependence was first studied in a multi-component buffer solution (“Polymix” buffer), which is characterized by the same buffer capacity in a wide pH range. During the work, we received biosensor responses to 1.5 mM L-arginine in the pH range of 5.03–9.11. According to the obtained dependence (Figure 2a), the highest sensitivity of the biosensor in the “Polymix” composition buffer was observed at pH 5.35. Considering that the further routine use of the biosensor should take place in a less complex buffer, we chose phosphate buffer solution as a potential one for use. Due to the fact that phosphate buffer has low buffering properties at pH < 6.0, a buffer solution with pH 6.2 was chosen for further measurements.

Another solution parameter that should be taken into account when working with conductometric biosensors is the buffer capacity. During the work, the dependence of the sensitivity of the biosensor to arginine on the concentration of the working buffer solution was investigated. For this purpose, calibration curves of the biosensor were obtained in a phosphate buffer solution at different concentrations (from 1 mM to 20 mM). The obtained results showed that the biosensor had the highest sensitivity to L-arginine in 1 mM phosphate buffer (Figure 2b, curve 1). At the same time, the operation of the biosensor in 1 mM and 2.5 mM buffer solutions was characterized by an increased level of signal noise compared to buffer solutions of a higher concentration, which strongly affected the accuracy of the signal amplitude measurement. This observation can be explained by the fact that the buffer properties of the solution cannot be preserved at low concentrations of salts. In general, from the obtained dependencies (Figure 2b), it was established that with an increase in the buffer capacity of the working solution, the response of the biosensor to the substrate decreased, and the linear range of the biosensor’s operation expanded. For further work, 5 mM phosphate buffer solution was chosen, as it simultaneously provides high sensitivity of the analysis and a convenient linear range of operation.

**Figure 3:**
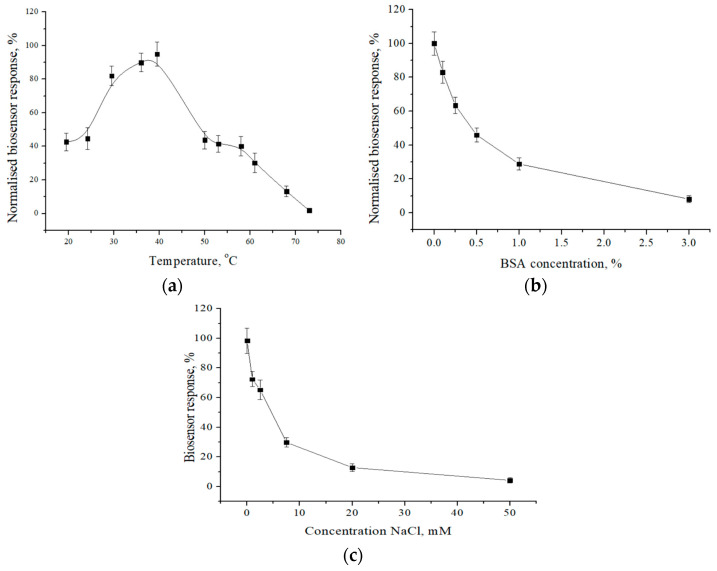
Dependence of the response of the biosensor on the temperature (**a**), protein concentration (**b**), and ionic strength (**c**) of the solution in the measuring cell. The concentration of L-arginine is 1.5 mM. Measurements were carried out in 5 mM phosphate buffer with pH 6.2.

In addition, it is well known that the speed of enzymatic reactions (namely the formation of a substrate–enzyme complex) highly depends on the temperature. Therefore, it is necessary to study the influence of the temperature of the working solution on the functioning of the developed biosensor. It was shown that the proposed ADI-based biosensor is sensitive to buffer solution temperature changes. Initially, when the thermal condition increased from room temperature to 39.5 °C, the response to arginine escalated by 54% (from the maximum response), and with a further growth in temperature, the response decreased sharply, which is associated with thermal denaturation of the protein part of the enzyme (with its subsequent destruction). The results of the measurements are shown in Figure 3a.

Almost all biological fluids contain a high concentration of protein molecules that can be adsorbed on the surface of the bioselective elements of the sensor and thus increase their density and, accordingly, impair their permeability to the substrate. It is obvious that due to the decrease in the amount of substrate that can penetrate the enzyme membrane of the biosensor, the response can decrease compared to a biosensor for which the working solution does not contain protein components.

To establish the nature of the dependence between the sensitivity of the biosensor and the amount of protein in the measuring solution, we provided a study using BSA as a model interferent. For this, we studied the dependence of the biosensor responses on the concentration of BSA in the working buffer solution in the range from 0% to 3% (Figure 3b). The resulting graph shows an inversely proportional relationship between the protein concentration in the solution and the response value of the biosensor. Thus, at a protein concentration of 3%, the initial response of the biosensor decreased by more than 90%. From this, we can conclude that the sensitivity of the biosensor largely depends on the presence of protein components in the measurement solution. Therefore, when using the biosensor to analyze samples that may potentially contain dissolved proteins, it is necessary to consider that such samples may require preliminary purification.

Another rather important parameter of the solution that can affect the performance of conductometric biosensors, especially when analyzing arginine in real samples, is the ionic strength of the solution. It (as well as the background conductivity of the solution) can increase with an increase in the buffer capacity. When analyzing real samples, the ionic strength may also change depending on the introduction of the test sample (in our case, food products and complex dietary supplements, which include substances that can cause uninformative changes in the electrical conductivity of the solution). Therefore, it was important to investigate exactly how the ionic strength affects the performance of the biosensor. During the experiment, the sensitivity of the biosensor to L-arginine was investigated using a phosphate buffer solution containing NaCl in concentrations from 0 mM to 50 mM. From the obtained data (Figure 3c), it can be seen that as the ionic strength of the solution increases, the sensitivity of the biosensor to L-arginine decreases. This can be explained by the influence of the background conductivity of the solution, characteristic of solutions of strong electrolytes, in which an increase in the concentration of the electrolyte leads to a decrease in the mobility of ions and the equivalent electrical conductivity of the solution. Therefore, it is extremely important to control the ionic strength of all working solutions used in the operation of conductometric biosensors, especially during the analysis of real samples.

### 3.2. Analysis of the Main Analytical Characteristics of the Conductometric Biosensor Based on Arginine Deiminase

**Figure 4:**
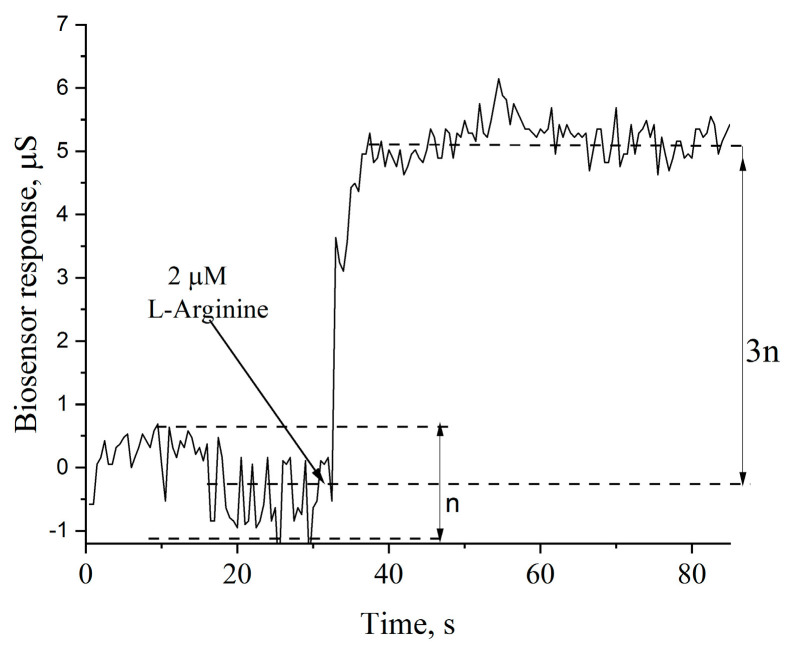
An example of a real biosensor response to the addition of a small concentration of analyte and the principle of the LOD calculation.

The next stage of creating a biosensor based on arginine deiminase was the study and generalization of its main analytical characteristics. For this purpose, a typical response of the biosensor at 1.5 mM was first obtained and its main parameters were analyzed (Figure 4). By analyzing the baseline before the response, noise and signal drift could be determined. Next, the minimum detection limit (limit of detection (LOD)) of the biosensor could be calculated. This parameter was calculated based on the typical response and then confirmed experimentally as the minimum arginine concentration at which the biosensor response exceeds three times the baseline noise level. Thus, the minimum detection limit was 2 µM. A response time of 1–1.5 min was also determined, while the duration of the entire analysis procedure was only 8 min.

**Figure 5:**
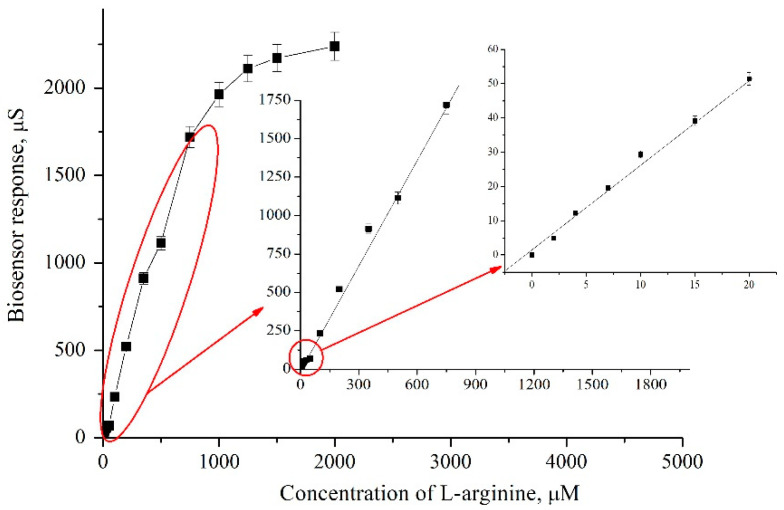
Calibration curve with an indication of its linear section. Measurements were performed in 5 mM phosphate buffer, pH 6.2.

For further study of the parameters of the biosensor for the analysis of L-arginine in model solutions, a detailed calibration curve was constructed (Figure 5). Theoretically, the linear section of this calibration curve can be used further to analyze the concentration of arginine in real samples.

The constructed calibration curve was analyzed, and the dynamic range of arginine analysis (up to 2000 µM) was determined. In turn, the linear operating range of the ADI-based biosensor was in the range from 2.5 to 750 μM arginine and was described by the following equation:

G = 4.396 + 2.273 * C,(2)

* note: G is the response of the biosensor to the addition of arginine to the measuring cell (μS), and C is the concentration of L-arginine (μM), R^2^ = 0.995.

In order to successfully study the necessary analytical characteristics of the newly developed biosensor, it is necessary to study its stability of operation and storage. In our previous work [40], which was devoted to the initial development of an ADI-based biosensor, we did not delve into the detailed optimization of the biosensor for the determination of arginine in real samples, but some basic parameters of the biosensor were verified. For example, signal reproducibility of the ADI-based biosensor during continuous operation was performed by monitoring its responses to the same concentration of arginine (1.5 mM) over several hours. It turned out that the relative standard deviation of the biosensor signals during the experiment was only 6.1% (n = 12). It was also previously checked how the sensitivity of the biosensor to arginine changes during long-term storage under different conditions. The stability of the biosensor during storage was studied for 31 days. Based on the obtained data, it was established that the biosensor based on ADI is best stored at a temperature of −18 °C. In these conditions, the biosensor was more stable than in others (80% of the initial activity of the sensor remained) after a month of storage.

**Figure 6:**
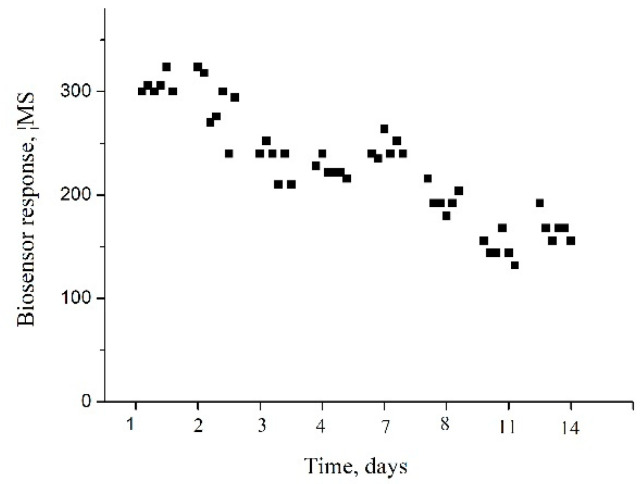
Operational stability of the biosensor ADI-based biosensor during 14 days of use. Biosensor responses to 1.5 mM L-arginine in 5 mM phosphate buffer solution, pH 6.2.

The stability of the biosensor was studied, which is also one of the main characteristics of its successful functioning. For this purpose, 6–7 responses to the same substrate concentration were obtained every day for two weeks (Figure 6). Between measurements, the biosensor was stored in a dry state at +8 °C. As can be seen from Figure 6, the biosensor was characterized by acceptable stability of operation during the first three days of use; after the first week of operation, the average response value of the biosensor decreased by 20%, and after the end of the experiment (after two weeks), by 45%. In general, it can be considered that the developed biosensor has good stability of operation during long-term use, especially since for the purpose of the quantitative determination of arginine, before each use, the calibration of the biosensor, which has already been in operation before, is carried out, which ensures the accuracy of the measurement results.

One of the most important criteria for the prospective use of the developed biosensor in the analysis of real samples is its target analyte selectivity relative to possible interferents. For this point, the sensitivity of the developed biosensor to a number of amino acids was studied in comparison with its sensitivity to arginine (Table 1). The response of the biosensor was calculated as a percentage, while 100% was taken as the response to the same concentration of L-arginine.

This study showed that the biosensor did not exhibit a response to most of the proteinogenic amino acids, with the exception of L-lysine and aspartic and glutamic acids; responses of the biosensor to the above-mentioned compounds were up to 20% of its response to L-arginine. In our opinion, these responses are related to a possible uninformative change in the conductivity of the investigated solution, caused by the limitations of the differential measurement mode. Since the addition of highly charged molecules can cause a significant increase in the background conductivity of the solution, which cannot be compensated for when the critical limit is exceeded, it can be reflected in the form of a biosensor signal.

In addition, the sensitivity of the biosensor to some electroactive substances that may be present in food products, namely to ascorbic and citric acids, was tested. The value of the response of the biosensor to citric acid was 133.3%, and to ascorbic acid, it was 53.3% relative to the response of the biosensor to the same concentration of L-arginine. Obviously, such responses of the biosensor are also uninformative and are caused by the limitations of the differential measurement mode, since these organic acids cause a significant increase in the background conductivity of the solution under investigation.

Since the presence of these acids in real samples can give a non-specific overestimation of the result of the analysis when determining the concentration of L-arginine, it is important to control the presence of these substances before setting up the analysis. If they are available, we consider it advisable to use additional membranes on the surface of the conductometric transducer, which would serve as a mechanical barrier for the penetration of such substances and thereby minimize their contribution to the background conductivity of the nearby environment. and electrode layer of the solution. In our opinion, one of the possible substances for use as a membrane can be polyphenylenediamine, which in our previous works made it possible to significantly increase the selectivity of amperometric electrodes [41].

After analyzing the responses, calibration curve, stability, and selectivity of the biosensor obtained in the working solution of the optimized composition, all the main analytical characteristics of the biosensor were collected in Table 2.

The limit of detection for L-arginine was measured as the minimum substrate concentration that gave a response greater than three times the background noise. Thus, the minimum detection limit was 2 µM. The linear operating range of the biosensor is from 20 to 750 μM L-arginine, and the dynamic range is from 0 to 2000 μM.

The baseline noise for this measurement was 0.72 µS/min, respectively.

### 3.3. Determination of Arginine in Real Samples Using the ADI-Based Conductometric Biosensor

The approbation of the conductometric biosensor based on arginine deiminase during the analysis of real samples was checked by determining the content of L-arginine in three types of arginine-containing dietary supplements, namely in “L-arginine” capsules produced by “Elit-Pharm” LLC (Dnipro, Ukraine), “L-arginine 500 mg” capsules produced by “Now Foods” (USA), and “L-arginine 500 mg” capsules produced by “Solgar Vitamin and Herb” (USA). Before analysis, the contents of each capsule were dissolved in 5 mM phosphate buffer solution (pH 6.2). To determine the unknown concentrations of arginine in the samples, three biosensor methods were tried (see Section 2.4.1). To select the best biosensor method for determining arginine in the “Solgar Vitamin and Herb” sample, five series of measurements were carried out using all three methods (Table 3).

Three methods are used to determine arginine in real samples. The first one is based on the use of a previously constructed calibration curve of the biosensor (“According to the calibration curve” in Table 3). The second is based on standard additions where three consecutive aliquots of known arginine concentrations are added to the measurement after sample addition and the resulting responses are plotted to extrapolate the initial sample concentration (“Standard Additions” in the table). For the latter (“By Proportion” in the table), in this method, the response of the biosensor to the real sample is compared with the response to a model solution of arginine.

As can be seen from the table, the method using the calibration curve turned out to be the most inaccurate when compared with the data declared by the manufacturer. This happens because this method does not take into account the influence of the matrix of the real sample. The rapid method by proportion, as expected, was the fastest and had good accuracy, although the measurement error was the largest.

For further experiments, it was decided to use the method of standard additions, because at the initial stage of the study, the results of arginine determination using this method showed a good correlation with the manufacturer’s data, and the measurement error was within the normal range. The only thing that had to be controlled when using the standard addition method was that all responses were within the linear range of the sensor.

**Figure 7:**
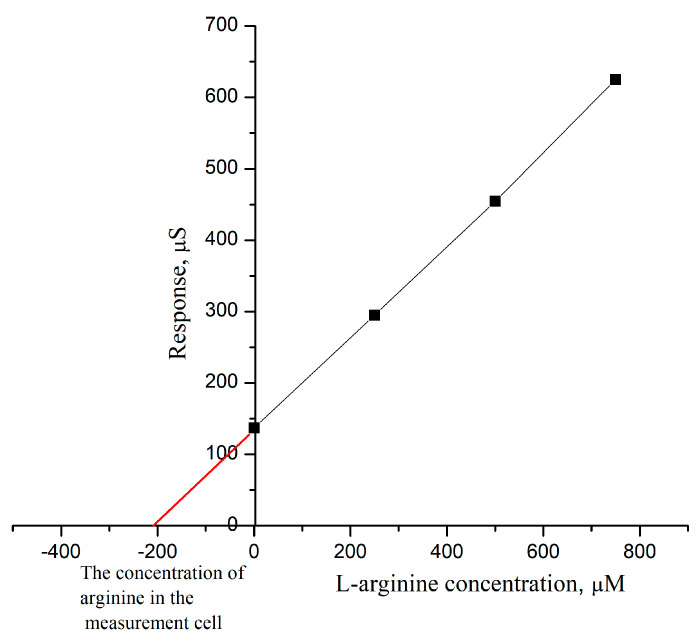
Calculation of L-arginine concentration in the measuring cell using the method of standard additions.

Accordingly, when conducting all subsequent experiments on the analysis of real samples using the method of standard additions, the response of the biosensor was first obtained on an aliquot of the studied sample; it was a sample of “Solgar Vitamin and Herb”. Firstly, it was diluted in 20 mL of water, and after that, it was ready for the measurement of the arginine concentration. For the measurement, firstly, into the measuring cell, 4 µL of sample solution was added. Then, responses were obtained based on three additions of L-arginine stock solution with a concentration of 250 µM each (Figure 7). In this way, four consecutive responses were obtained, on the basis of which the dependence was built in such a way that the response of the biosensor to the sample under study was plotted on the Oy axis (black line on Figure 7). A linear extrapolation of this graph intersected the Ox axis at a point that corresponded to the concentration of L-arginine in the measurement cell (red line on Figure 7). After multiplying the concentration of the sample in the measurement cell by the dilution factor, the concentration of L-arginine in the sample was obtained. Measurements for each sample were performed in five repetitions.

**Figure 8:**
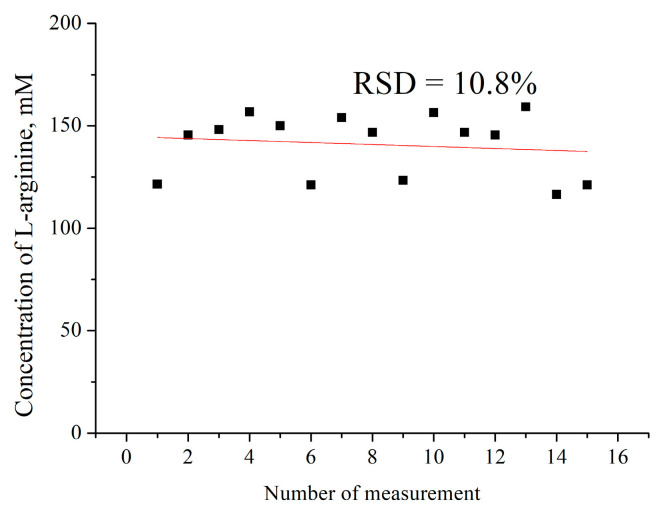
Repeatability of results of measurements in real samples via biosensor-based ADI. Measurement in 5 mM phosphate buffer, pH 6.2 (method of standard additions (500-fold dilution)).

It is known that when biosensors work with real samples, the reproducibility of the results deteriorates in comparison with work in model samples. Accordingly, it was necessary to investigate how the RSD of the biosensor’s work will change with the determined concentration of arginine in the pharmaceutical samples. For this purpose, a series of biosensor analyses of the arginine concentration in one sample of “Solgar Vitamin and Herb” was carried out during one working day. Determination of the concentration of arginine in a real sample was carried out 15 times with the same biosensor. The relative standard deviation between measurements was about 10.8%. Of course, in this case, the repeatability is much worse than when testing model samples (RSD = 6.1%), but this is a well-known effect for real analyses, in particular pharmaceutical samples (Figure 8).

**Figure 9:**
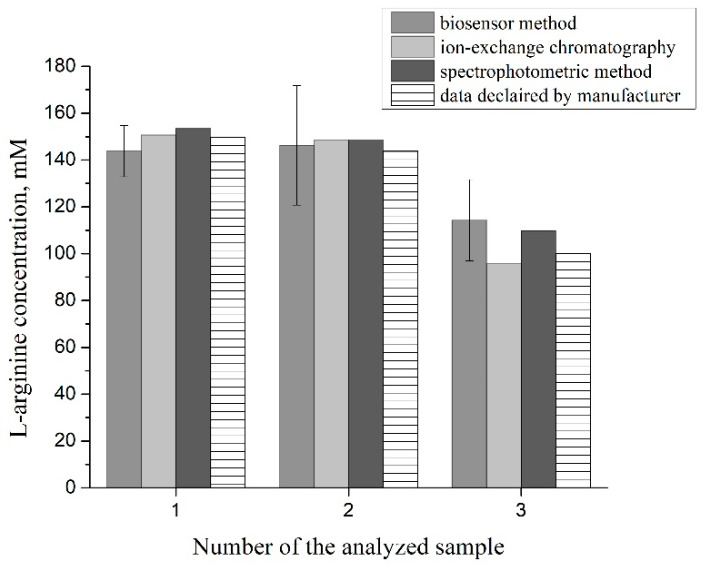
Comparison of L-arginine determination data in dietary supplement samples obtained using the biosensor method (n = 5) and control methods. Samples of dietary supplements: 1—“L-arginine 500 mg” (“Solgar Vitamin and Herb”); 2—“L-arginine 500 mg” (“Now Foods”); 3—“L-arginine” (LLC “Elit-Pharm”).

To control the biosensor results and the data provided by the manufacturer, the content of arginine in the samples was also determined using two control methods, the ion-exchange chromatography method (conducted by an independent laboratory of the Expert Center for Diagnostics and Laboratory Support “Biolights” LLC (Kyiv)) and using the spectrophotometric method (performed using the reagents and methods of the MAK370 commercial kit, “Sigma-Aldrich”). The results of the biosensor determination of arginine in capsules of dietary supplements in comparison with the results of control methods and the data declared by the manufacturers are shown graphically in Figure 9.

**Figure 10:**
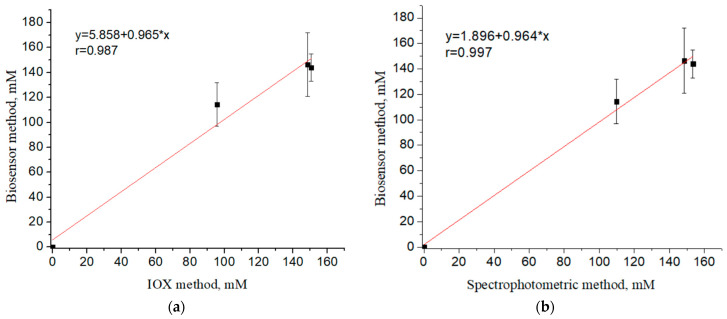
Correlation between data of the biosensor determination of L-arginine content in samples of dietary supplements and data of ion-exchange chromatography (**a**) and data of the spectrophotometric analysis (**b**). Biosensor determination was carried out in 5 mM phosphate buffer, pH 6.2.

Based on the calculated correlation coefficients (Figure 10), it was established that the results of determining L-arginine using a biosensor based on arginine deiminase were well correlated both with the data obtained using the ion-exchange chromatography method and with the results of the spectrophotometric method; correlation coefficients were r = 0.987 and r = 0.997, respectively. Indirectly, the almost perfect correlation obtained among all methods indicates that the concentrations of arginine in dietary samples are correctly specified by the manufacturers.

At the next stage of this study, the influence of various sample preparation procedures on the measurement results was detected. The research was conducted for one of the samples, namely for the dietary supplement “L-arginine 500 mg” manufactured by “Solgar Vitamin and Herb”. We compared the results of the measurement in the sample without preliminary sample preparation (only the transfer of the contents of the capsule into the solution) and after the following options for processing the sample in order to remove compounds that may affect the accuracy of the arginine determination: (1) dissolving the contents of the capsule followed by filtering the obtained solution through 1 µm and 0.22 µm filters (Cat. No. 729228, Chromafil Xtra, Macherey-Nagel, Germany and Cat. No. CE 0459, Millex GP, Merck Millipore, Ireland, respectively) and (2) centrifugation of the unfiltered sample at 2800 rpm within 10 min. The results of the biosensor determination of arginine in the sample after sample preparation are presented in Table 4.

The obtained data allow us to conclude that filtering the sample before the analysis leads to pre-concentration of the sample and, thus, to an overestimation of the arginine measurement result. Comparing the data of the biosensor determination for an unfiltered sample and the results of chromatographic analysis, it can be assumed that the dilution of the sample without its subsequent filtering is sufficient to obtain a reliable result of the analysis.

It is also worth noting that centrifugation of the unfiltered sample was also an unnecessary technique, as it led to some underestimation of the measurement result, apparently due to the extraction of a certain amount of L-arginine from the solution being analyzed. From the conducted study, it can be concluded that the use of the developed biosensor for the analysis of capsules of dietary supplements does not require additional procedures for preparing samples for analysis in order to extract excipients, which gives an advantage to the biosensor method of analysis in comparison with traditional analytical methods.

### 3.4. Comparison of Analytical Characteristics of ADI-Based Biosensor with a Previously Developed Biosensor for Arginine Determination

A comparison of the analytical characteristics of the developed biosensor with other electrochemical biosensors for arginine determination reported in the literature over the past 15 years (Table 5) reveals that the proposed conductometric biosensor based on ADI possesses a superior set of operating parameters. Notably, this biosensor has one of the lowest detection limits (2 μM), a broad linear range (2.5–750 μM), and one of the highest storage stability rates, which are crucial for real sample analysis and potential commercialization. Also, due to the monoenzyme bioselective element our developed biosensor is one of the most selective ones.

## 4. Conclusions

The present study reports on the development, adaptation, and optimization of a new monoenzyme conductometric biosensor for the determination of arginine in dietary supplements. This biosensor is based on a recombinant arginine deiminase (ADI) from Mycoplasma hominis and is designed to accurately determine arginine levels in dietary supplements. The biosensor was created by immobilizing ADI on the sensitive surface of the conductometric transducer’s interdigitated electrodes, utilizing the glutaraldehyde-mediated crosslinking technique.

To achieve the highly sensitive detection of arginine in real samples, we investigated the impact of various parameters (such as pH, buffer capacity, ionic strength, temperature, and BSA concentration) on the biosensor’s sensitivity towards arginine. Subsequently, we examined the analytical characteristics, signal reproducibility, and storage stability of the biosensor in the buffer solution with the optimized composition. The biosensor was characterized by good reproducibility of results (for 12 measurements, the RSD was 6.1%) and operational stability, retaining 55% of the initial response after two weeks of continuous use. Moreover, it demonstrated good reproducibility during long-term storage; after one month of dry state at a temperature of −18 °C, the response was 80% of the initial one.

The concentration of arginine in pharmaceutical samples was determined using the method of standard additions, as it was found to be the most effective and accurate detection method during the experimental work. The selection of the sample pretreatment method for the measurement was performed, and it was determined that the most optimal approach is the dissolution of the sample capsule contents in distilled water without further filtration or centrifugation.

It was established that the results of determining L-arginine using a biosensor based on arginine deiminase were well correlated both with the data obtained using the ion-exchange chromatography method and with the results of the spectrophotometric method; correlation coefficients were R = 0.987 and R = 0.997, respectively.

We believe that the conductometric biosensor based on arginine deiminase from M. hominis can be considered a promising analytical tool for the highly sensitive determination of L-arginine in the wide spectrum of real samples due to the small size, high-selective and high-sensitive performance, ease of use, and a possibility of usage in different places due to the portability of the device.

## Figures and Tables

**Table 1:** Selectivity of the biosensor based on arginine deiminase relative to basic amino acids. Study of the selectivity of a biosensor based on arginine deiminase. Study of biosensor responses to each of the amino acids.

Amino Acids (100 µM)	Relative Response of the Biosensor, %
Arginine	100.0 ± 6.2
Aspartic acid	20.0 ± 2.7
Glutamic acid	20.0 ± 4.0
Lysine	6.7 ± 1.0
Methionine, asparagine, glutamine, histidine, serine, valine, alanine, tryptophan, phenylalanine, proline, threonine, glycine, isoleucine, cysteine	0.0 ± 0.0

**Table 2:** Main analytical characteristics of the conductometric biosensor based on arginine deiminase.

Analytical Characteristic of Biosensor, Units	Response
Linear range, µM	2.5–750.0
Dynamic range, µM	0.0–2000.0
Limit of detection, µM	2.0
Baseline noise, µS/min	1.6
Response time, min	1.0–1.5
Total time of one analysis, min	8.0
Residual activity after a month of storage at −18 °C, %	80.0
RSD of response, %	6.1
Residual activity after 2 weeks of operational stability study, %	55.0

**Table 3:** The influence of the biosensor measurement method on the result of determining the concentration of L-arginine in real samples.

Method of Determination Measurement Number	“Standard Additions”, mM	“By Proportion”, mM	“According to the Calibration Curve”, mM	Declared by the Manufacturer, mM
1	125.00	125.00	195.35	149.70
2	153.57	138.16	203.11	
3	147.83	164.06	201.25	
4	146.43	145.80	204.13	
5	146.48	154.14	207.45	
**Mean ± SD**	**143.86 ± 10.95**	**145.43 ± 14.94**	**202.25 ± 4.47**	

**Table 4:** Influence of different sample preparation procedures on the results of the determination of L-arginine in dietary supplement capsules.

	Sample Preparation Procedure			Declared by the Manufacturer
	Sample Filtering	Centrifugation of the Unfiltered Sample	Unfiltered Sample	
Concentration of L-arginine, determined by biosensor, mM	173.61 ± 29.10	135.87 ± 28.37	143.86 ± 10.95	149.70

**Table 5:** Analytical characteristics of the developed biosensor in comparison with previously developed ones.

#	Recognition Element	Type of Transducer	Limit of Detection, µM	Linear Range, µM	Stability	Real Samples Measurement	Reference
1	Urease and recombinant arginase I	Potentiometic	500	500–1700	Approximately 14 days when stored in +4 °C	Pharmaceuticals	[31]
	Urease, recombinant arginase I, and gold nanoparticles	Potentiometric	120	120–1700			
2	Urease and recombinant arginase I	Amperometric (Nafion/PANi-modified electrode)	38	70–600	Residue activity 20% after 24 h (half-life of the biosensor was about 72 h)	Pharmaceuticals	[30]
3	Urease and boline liver arginase	Conductometric	0.5	10–4000	Residual activity 90% after 45 days	Pharmaceutics	[34]
4	Urease and recombinant Hansenula polymorpha cells over-producing human liver arginase I	Amperometric	85	15–600	Residue activity 80% after 24 h (half-life of the biosensor was about 72 h)	Wine and juice	[32]
5	Urease and recombinant human arginase I	Potentiometric	up to 0.001	0.001–100,000	Storage stability about 31 days	Juice	[42]
6	Recombinant arginine deiminase	Amperometric (Nafion/PANi-modified electrode)	1	3–200	Residue activity 93% after 35 days of storage (100 assays)	Pharmaceuticals and human plasma	[43]
7	Urease and bovine liver arginase	Potentiometric	10	10–1000	n/a	n/a	[33]
8	Urease and bovine liver arginase	Conductometric	2.5	2.5–500	n/a	Pharmaceuticals	[29]
9	Pyrene methyl ammonium chloride and polystyrene sulfonate	Ratiometric optical	35	0–1600	n/a	n/a	[44]
10a	L-arginine oxidase and nanoparticles of CeCu	Amperometric	n/a	5–100	Residual activity about 50% after 5 days—10a and 10b, 10c—50% after 3 days	Pharmaceuticals, juice and wine	[26]
10b	L-arginine oxidase and nanoparticles of NiPtPd		n/a	10–250			
10c	L-arginine oxidase and green hexacyanoferrate of copper		n/a	10–100		n/a	
11	Recombinant arginine deiminase from Mycoplasma hominis	Conductometric	2	2.5–750	Residual activity 80% after 31 days in −4 °C.	Pharmaceuticals	Current work
					Repeatability of results—RSD = 6.1%		

## Data Availability

Data can be provided upon request.
